# Primary pelvic hydatidosis associated with ovarian attachment: A case report

**DOI:** 10.1093/omcr/omaf279

**Published:** 2025-12-26

**Authors:** Melody Omrani Nava, Majidreza Adelani, Samie Alinejad, Nazanin Mirzaei, Ghazal Goli, Eissa Soleymani

**Affiliations:** Department of Infectious disease, Assistant Professor, Hospital administration research center, Sari, Islamic Azad University, Sari 48164-194, Iran; Department of Internal Ward, Pulmonary and Critical Care Division, Mazandaran University of Medical Sciences, Sari 4815733971, Iran; Department of Gynecology, Ramsar Imam Sajjad Hospital, Mazandaran University of Medical Sciences, Mazandaran, Sari 4815733971, Iran; Department of Clinical Pathology, Islamic Azad University, Tonekabon Branch, Tonekabon 4684161167, Iran; Department of Gynecology, Juybar Hajazizi Hospital, Mazandaran University of Medical Sciences, Sari 4815733971, Iran; Toxoplasmosis Research Center, Communicable Diseases Institute, Mazandaran University of Medical Sciences, Sari, Iran; Department of Parasitology, Faculty of Medicine, Mazandaran University of Medical Sciences, Sari, Iran

**Keywords:** hydatid cyst, pelvic, Hydatidosis, *Echinococcus granulosus*

## Abstract

**Background:**

Hydatid cyst (HC) represents a remarkable zoonotic parasitic disease, mostly affecting the liver and lungs. In this report, we present a patient from northern Iran who exhibited HC involvement in the pelvic region, a rare occurrence that is often asymptomatic.

**Case Presentation:**

A 41-year-old female presented with hypogastric pain, and imaging survey showed the potential presence of cystadenoma, endometriosis, and various typical ovarian lesions. She underwent laparotomy, during which pelvic HC was identified. Serological testing confirmed this finding. Also, albendazole was prescribed, and she was discharged in relatively good condition.

**Conclusion:**

In regions where this infection is prevalent, HC must be considered in the differential diagnosis of ambiguous cystic tumors. This case highlights the importance of including HC in the differential diagnosis of pelvic cysts, especially in endemic areas. Furthermore, we recommend the using of molecular techniques during surgical interventions as potentially beneficial.

## Introduction

Hydatid cyst (HC) is a zoonotic infection caused by the larval stage of *Echinococcus granulosus* (*E. granulosus*) [1]. In the small intestines of carnivorous animals, such as dogs, which serve as the definitive host for the parasite, the eggs are excreted in the feces of these animals [2]. Subsequently these eggs are then accidentally ingested by humans and upon ingestion, the larvae penetrate the intestinal wall and spread throughout the body via the bloodstream [1, 2]. While HC is a rare infection in the world but its prevalence in Iran is relatively high [3]. The average prevalence of HC in northern Iran, due to the presence of extensive traditional animal husbandry, is high (almost 9%), making it one of the most contaminated areas of Iran [2, 3].

All organs have possibility of HC infection and the liver and lung are most common site and other organs are affected rarely [4]. The incidence of HC in the pelvis is rare and does not have special sign and patient’s pathology is diagnosed during or after surgery [5]. Based on lesion location, surgical interventions and rarely medical treatment is preferred. Finding reported cases of HC in the pelvis is very valuable because it is very uncommon and its diagnosis is difficult [5]. Here, we would like to introduce a patient with pelvis hydatidosis from northern Iran.

## Case report

A rural 41-year-old woman from northern Iran has experienced hypogastric pain for about one year prior to her presentation, with the pain aggravating during walking. During the physical examination, the abdomen was found to be soft, without tenderness or organomegaly, and no skin lesions were noted. The bimanual pelvic examination revealed the presence of a mobile mass measuring 60 × 80 × 40 mm located in the right pelvic wall and sacrum. The uterus was of normal size, with the parametrium on both sides and the rectal mucosa appearing unremarkable. All laboratory tests yielded normal results. The ultrasound examination showed that the left ovary measured 22 × 30 mm and exhibited a normal echo pattern. In the right adnexa, the right ovary also measured 22 × 30 mm; but a cyst measuring 72 × 60 mm was observed, characterized by coarse echoes, a smooth wall, and multiple septations.

**Figure 1:**
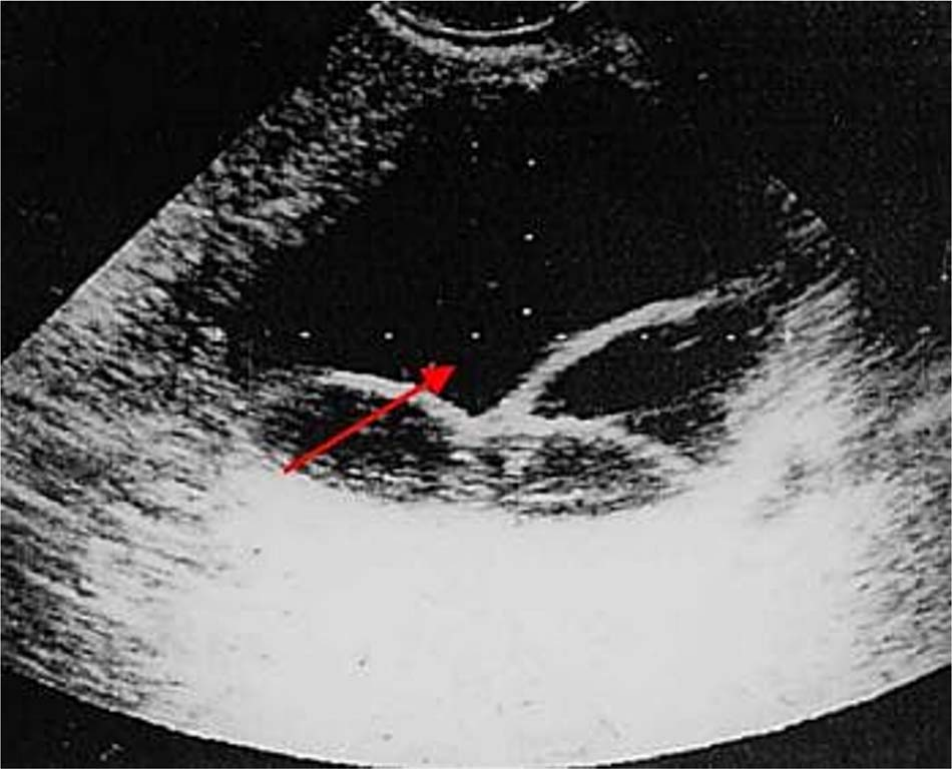
Ultrasound view of ovary of patient whit pelvic HC in the northern Iran.

**Figure 2:**
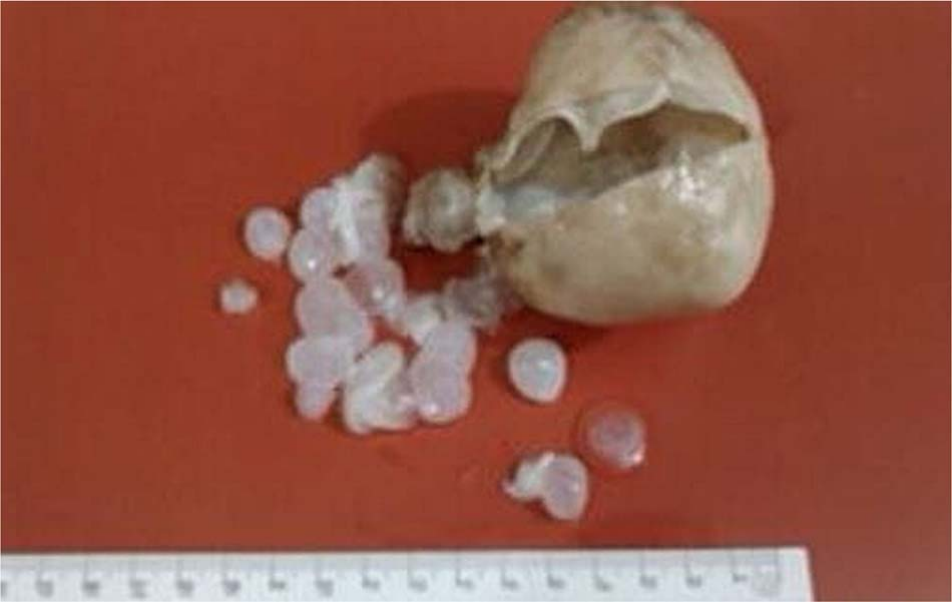
Pelvic HC removed from a patient in the north of Iran.

The ultrasound findings suggested the possibility of cystadenoma, endometriosis, and other prevalent ovarian lesions (Fig. 1). The patient, suspected of having an ovarian cyst, underwent a laparotomy and right salpingo-oophorectomy through a midline incision. The surgical report showed the presence of a 30 mm cyst in the right adnexa, along with an 80 mm mass situated posterior to the uterus, which was adherent to both the intestine and peritoneum. The mass was excised in its entirety, and the patient subsequently underwent a right salpingo-oophorectomy. The mass was then sent for pathology ward (Fig. 2). The pathology report identified cysts with a fibrotic wall, characterized by an outer laminated elastic layer and an inner germinal layer that exhibited numerous papillae containing scolices, suggestive of a HC. Following this, an enzyme-linked immunosorbent assay (ELISA) for *E. granulosus* yielded a positive result. Postoperatively, albendazole (400 mg twice daily) was prescribed for 3 months. The patient was discharged with relatively good satiation. She showed no recurrence during 6-month monthly follow-up.

## Discussion

This report delineates a clinically remarkable case of primary pelvic HC in a rural woman from northern Iran, an area recognized as highly endemic for HC. The primary clinical and ultrasonographic appearance was highly suggestive of a benign ovarian neoplasm, such as a cystadenoma or an endometrioma. This diagnostic problem is a recurring theme in cases of pelvic HC, as the imaging features are notoriously nonspecific and may simply be mistaken for more Common adnexal masses [5, 6]. Ultrasonography, which is acknowledged as the primary imaging technique for gynecological assessments, shows low sensitivity in diagnosing HC of the pelvis and ovaries [3]. However, high-frequency transvaginal ultrasound, operating at 7.5 MHz, has been proposed as an useful method for identifying HC in this region [7]. In our case, this was also true, as the HC was found to be attached with the ovary, and ultrasound did not assist in the preoperative diagnosis of the infection; and postoperative diagnosis was confirmed via pathology.

The following serological approval by ELISA confirmed the diagnosis, of course it is well-documented that serological tests may yield false-negative finding, especially in cases with low antigenic load [1, 2]. In the present case, the cyst was removed by laparotomy completely, and albendazole was also prescribed. In a report by Ranzini reviewing 20 388 laparotomies and 1463 hysterectomies indicated that only 0.028% of cases involved genitopelvic hydatidosis [8]. The infection of the pelvis by HCs presents considerable risks due to its asymptomatic nature, which allows for the parasitic infiltration of the sacroiliac and hip joints [9]. Furthermore, complications associated with HC may include the stimulation of malignancy and limitation during labor [8]. Even a case of severe reported that HC affecting the pelvis and femur, which required a hindquarter amputation [10].

HC should be regarded as a possible diagnosis when addressing atypical pelvic cystic masses, especially in patients from endemic areas [6]. The lack of hepatic or pulmonary involvement challenges the typical understanding of hydatidosis, emphasizing the genital tract as a primary site, a situation that is rarely reported [3, 5]. While the majority of these cases are secondary [5, 9, 10], our case was primary involvement. Pelvic localization of HC is very rare, even in endemic areas [1, 4]. Primary pelvic HC is thought to occur via hematogenous spreading. Rural residence and the high prevalence of *E. granulosus* in the livestock of northern Iran [1, 3] are important epidemiological reasons that predisposed individuals to HC. In certain cases, the presence of pelvic HC can even lead to infertility [5]; however, this was not applicable in our case.

The absence of molecular method, such as cell-free DNA (CFD), which is a highly specific molecular tool for detecting *Echinococcus* DNA, represents an ignored gap. The preoperative application of CFD could have facilitated diagnosis, allowing for more precise surgical planning [5]. we recommend all gynecologists and radiologists should maintain a heightened suspicion for HCs whenever a cystic pelvic mass is detected [1, 7]. In our case, the adherence of the cyst necessitated a salpingo-oophorectomy to attain complete removal. The administration of albendazole was a standard adjunctive therapy aimed at reducing the viability of protoscolices, thereby minimizing the risk of secondary recurrence from potential microscopic spillage [7]. The patient’s good conditions, with no recurrence, validated this combined surgical and pharmacological approach.

## Conclusion

Our case highlights the importance of including HC in the differential diagnosis of cystic pelvic masses. We propose that the use of molecular techniques in surgical procedures of HCcan very beneficial. Furthermore, we recommend all gynecologists and radiologists should maintain a heightened suspicion for HCs whenever a septate cystic pelvic mass is detected, particularly in endemic areas.
